# The Burden and Impact of Early Post-transplant Multidrug-Resistant Organism Detection Among Renal Transplant Recipients, 2005–2021

**DOI:** 10.1093/ofid/ofae060

**Published:** 2024-02-13

**Authors:** Ahmed Babiker, Geeta Karadkhele, Andrei Bombin, Rockford Watkins, Chad Robichaux, Gillian Smith, Vivek B Beechar, Danielle B Steed, Jesse T Jacob, Timothy D Read, Sarah Satola, Christian P Larsen, Colleen S Kraft, Stephanie M Pouch, Michael H Woodworth

**Affiliations:** Division of Infectious Diseases, Department of Medicine, Emory University School of Medicine, Atlanta, Georgia, USA; Department of Pathology and Laboratory Medicine, Emory University School of Medicine, Atlanta, Georgia, USA; Emory Transplant Center and Department of Surgery, Emory University School of Medicine, Atlanta, Georgia, USA; Division of Infectious Diseases, Department of Medicine, Emory University School of Medicine, Atlanta, Georgia, USA; Division of Infectious Diseases, Department of Medicine, Emory University School of Medicine, Atlanta, Georgia, USA; Department of Biomedical Informatics, Emory University School of Medicine, Atlanta, Georgia, USA; Division of Infectious Diseases, Department of Medicine, Emory University School of Medicine, Atlanta, Georgia, USA; Georgia Emerging Infections Program, Atlanta, Georgia, USA; Atlanta Veterans Affairs Medical Center, Atlanta, Georgia, USA; Division of Infectious Diseases, Department of Medicine, Emory University School of Medicine, Atlanta, Georgia, USA; Division of Infectious Diseases, Department of Medicine, Emory University School of Medicine, Atlanta, Georgia, USA; Division of Infectious Diseases, Department of Medicine, Emory University School of Medicine, Atlanta, Georgia, USA; Division of Infectious Diseases, Department of Medicine, Emory University School of Medicine, Atlanta, Georgia, USA; Division of Infectious Diseases, Department of Medicine, Emory University School of Medicine, Atlanta, Georgia, USA; Emory Transplant Center and Department of Surgery, Emory University School of Medicine, Atlanta, Georgia, USA; Division of Infectious Diseases, Department of Medicine, Emory University School of Medicine, Atlanta, Georgia, USA; Department of Pathology and Laboratory Medicine, Emory University School of Medicine, Atlanta, Georgia, USA; Division of Infectious Diseases, Department of Medicine, Emory University School of Medicine, Atlanta, Georgia, USA; Division of Infectious Diseases, Department of Medicine, Emory University School of Medicine, Atlanta, Georgia, USA

**Keywords:** allograft failure, MDROs, renal transplant

## Abstract

**Background:**

Reducing the burden of multidrug-resistant organism (MDRO) colonization and infection among renal transplant recipients (RTRs) may improve patient outcomes. We aimed to assess whether the detection of an MDRO or a comparable antibiotic-susceptible organism (CSO) during the early post-transplant (EPT) period was associated with graft loss and mortality among RTRs.

**Methods:**

We conducted a retrospective cohort study of RTRs transplanted between 2005 and 2021. EPT positivity was defined as a positive bacterial culture within 30 days of transplant. The incidence and prevalence of EPT MDRO detection were calculated. The primary outcome was a composite of 1-year allograft loss or mortality following transplant. Multivariable Cox hazard regression, competing risk, propensity score–weighted sensitivity, and subgroup analyses were performed.

**Results:**

Among 3507 RTRs, the prevalence of EPT MDRO detection was 1.3% (95% CI, 0.91%–1.69%) with an incidence rate per 1000 EPT-days at risk of 0.42 (95% CI, 0.31–0.57). Among RTRs who met survival analysis inclusion criteria (n = 3432), 91% (3138/3432) had no positive EPT cultures and were designated as negative controls, 8% (263/3432) had a CSO detected, and 1% (31/3432) had an MDRO detected in the EPT period. EPT MDRO detection was associated with the composite outcome (adjusted hazard ratio [aHR], 3.29; 95% CI, 1.21–8.92) and death-censored allograft loss (cause-specific aHR, 7.15; 95% CI, 0.92–55.5; subdistribution aHR, 7.15; 95% CI, 0.95–53.7). A similar trend was seen in the subgroup and sensitivity analyses.

**Conclusions:**

MDRO detection during the EPT period was associated with allograft loss, suggesting the need for increased strategies to optimize prevention of MDRO colonization and infection.

## Teaser

In a retrospective study of 3,507 renal transplant recipients (RTRs) the detection of an MDRO during the early post-transplant (EPT) period was associated with death-censored allograft loss. This suggests the need for increased strategies to optimize prevention of MDROs colonization and infection.

The World Health Organization (WHO) and Centers for Disease Control and Prevention (CDC) have declared antimicrobial resistance (AMR) a priority public health threat [1, 2]. Therapeutic options for patients with multidrug-resistant organism (MDRO) infections are limited, less efficacious, and more toxic compared with those for susceptible infections. For these reasons, rates of mortality are disproportionately increased when compared with infections caused by susceptible bacteria [3–5]. MDRO colonization, defined as the detection of an organism without signs or symptoms of infection, frequently precedes invasive clinical infection [6–8].

Renal transplant recipients (RTRs) represent a vulnerable population at increased risk of MDRO acquisition and colonization [9]. This is especially true in the early post-transplant (EPT) period due to prolonged hospitalization, the need for invasive procedures and indwelling devices, exposure to broad-spectrum antibiotics, and a higher net state of immunosuppression [9–12]. Post-transplant infection, including MDRO infection, is a major cause of mortality and has been linked to allograft loss among RTRs [3, 13, 14].

Understanding the impact of MDRO colonization and infection on RTR patient mortality and allograft function is paramount to mitigating these deleterious outcomes. Prior studies have been limited by lack of control groups, small sample sizes, a focus on 1 taxon/species of MDROs, or a single infection site (such as the urinary tract) [15–19]. To overcome previous limitations, we utilized a cohort of >4000 RTRs followed from 2005 to 2021 to estimate the prevalence and incidence of EPT MDRO detection among RTRs and compare mortality and allograft failure, among RTRs with EPT MDRO detection compared with a control group of RTRs.

## METHODS

### Study Setting, Design, and Data Sources

The Emory Transplant Center performs ∼250 adult renal transplants annually and provides ongoing care for >4000 renal transplant patients. We performed a retrospective cohort study of RTRs in the Emory Renal Transplant database [20] between 2005 and 2021. Data are prospectively entered into the database directly from Emory's electronic medical records. The study was approved by the Emory University Institutional Review Board.

### Study Participants

Adult RTRs undergoing their first episode of renal transplantation (including kidney-pancreas and kidney-liver) at Emory were included. RTRs <18 years old and/or who had their renal transplant performed outside of Emory University Hospital or who were undergoing a repeat renal transplant were excluded.

### Exposure, Outcomes, and Covariate Definitions

The EPT period was defined as 30 days from transplant. The exposure variable was defined as an EPT bacterial culture positive for a target MDRO or a comparative antibiotic-susceptible organism (CSO). EPT culture positivity was defined as a positive culture (irrespective of culture specimen site) within 30 days of renal transplant. MDRO selection was guided by the CDC and WHO MDRO priority lists and the American Society of Transplantation (AST) Infectious Diseases Community Guidelines [1, 2, 9, 21, 22]. These included methicillin-resistant *Staphylococcus aureus* (MRSA), vancomycin-resistant *Enterococcus* (VRE), extended-spectrum cephalosporin-resistant Enterobacterales (ESCRE), carbapenem-resistant Enterobacterales (CRE), carbapenem-resistant *Acinetobacter baumanii* complex (CRAB), and carbapenem-resistant *Pseudomonas aeruginosa* (CRPA). CSOs included methicillin-susceptible *Staphylococcus aureus* (MSSA), vancomycin-susceptible *Enterococcus* (VSE), extended-spectrum cephalosporin-susceptible Enterobacterales (ESCSE), carbapenem-susceptible *P. aeruginosa* (CSPA), and carbapenem-susceptible *A. baumanii* complex (CSAB) (Supplementary Table 1). Urine cultures obtained within the EPT period are obtained for cause as determined by the treating team and not in a routine systematic fashion. No systemic MDRO rectal screening of RTRs was performed during the study period. Antimicrobial susceptibility testing was performed at the Emory University Hospital microbiology laboratory as part of routine clinical care on the MicroScan (Beckman Coulter, Brea, CA, USA) initially and then on the Vitek 2 (bioMérieux, Durham, NC, USA) from 2017 onwards. MDROs and CSOs were defined as per Clinical and Laboratory Standards Institute (CLSI) breakpoints each year [23].

The primary outcome was a combined composite outcome of 1-year allograft loss or mortality following renal transplant. One-year allograft loss was defined as renal replacement therapy for >3 months without subsequent improvement or relisting for transplant within 1 year of renal transplant, and 1-year post-transplant mortality was defined as death within 1 year of renal transplant.

Analyzed covariates included sex, age at time of renal transplant, race (used as a surrogate for social–demographic effects rather than a biological variable), induction immunosuppressive regimen, etiology of end-stage renal disease (ESRD), type of renal transplant donor (living vs cadaveric donor), pretransplant positive MDRO culture status (any), dual solid organ transplant status (kidney-pancreas or kidney-liver), year of transplantation, CMV serostatus of donor and recipient, and biopsy-proven acute graft rejection. Antibiotic and nephrotoxic drug exposure were not included as covariates. All variables included were obtained from the electronic medical record (EMR).

### Exclusion Criteria

RTRs were excluded from the survival analysis if they had a positive EPT culture that did not meet MDRO/CSO criteria or had key susceptibility results missing (eg, vancomycin for *Enterococcus* spp.). To avoid misclassification bias, RTRs who had both MDROs and CSOs on culture in the EPT period were also excluded.

### Statistical Analysis

The prevalence of EPT MDRO detection was estimated using all eligible RTRs as the denominator. The incidence rate of EPT MDRO detection was calculated per 1000 EPT-days at risk (Supplementary Data). Differences in incidence rates pre- and postchange in CLSI breakpoint were assessed using the Wilcoxon signed-rank test. The overall change in incidence over time was assessed using a negative binomial model with adjustment for pre- and postchange in CLSI breakpoint, and model fit was assessed using a log likelihood test (compared with the model without breakpoint change).

To examine the impact of post-transplant MDRO detection on post-transplant outcomes, we examined the hazards of experiencing the primary outcome among 3 mutually exclusive exposure categories: (i) RTRs with EPT MDRO detection, (ii) RTRs with EPT CSO detection, (iii) RTRs with no positive cultures during the EPT period. RTRs with EPT MDRO and CSO detection were compared with a control group of RTRs with no positive cultures (negative control group). Cohort characteristics were described by overall and EPT culture positivity status. Among RTRs with multiple MDROs or CSO detected in the EPT period, the earliest post-transplant organism episode was included. A Kaplan-Meier survival analysis was performed, and differences between survival curves were assessed using the log-rank test. Multivariable Cox proportional hazard regression modeling was performed, with adjustment covariates selection informed by literature review, directed acyclic graph (Supplementary Figure 1), and bivariate analysis. None of the variables violated the proportional hazard assumption by graphical and Schoenfeld residual statistical methods. Given the competing risks of risk of death on allograft loss, a competing risk analysis was performed by fitting a cause-specific and subdistribution hazard model for both outcomes (mortality and allograft loss). The competing cumulative incidences of each outcome (mortality and allograft loss) by exposure group were compared using Gray's test, with the comparator group being the negative control group for both the MDRO and CSO groups. Wilcoxon tests were used to compare these measurements between the treatment groups. Adjusted hazard ratios (aHRs) and corresponding 95% CIs were reported. The following subgroup analyses were performed: (1) among patients with positive urine cultures (urine subgroup), (2) among patients in the post-Enterobacterales breakpoint change period (2011–2021) [24], (3) kidney transplant–only patients (removing those with kidney-pancreas or kidney-liver).

A sensitivity analysis was performed to adjust for the propensity of MDRO acquisition on outcomes. A propensity score (PS)–weighted analysis was performed where a PS was generated based on the probability of each exposure category stratum (Supplementary Data). After PS calculation, 2 methods of weighting that incorporated the PS, inverse probability of treatment weighting (IPTW) and matching weighting (MW), were applied to create weighted cohorts (Supplementary Figure 2) [25]. Weighted multivariable Cox proportional hazard modeling was performed using the MW and IPTW cohorts. Statistical analysis was performed with R using Rstudio, version 2022.12.0.353 (R Foundation for Statistical Computing, Vienna, Austria).

## RESULTS

### Prevalence and Incidence of Early Post-transplant MDRO Detection

**Figure 1.**
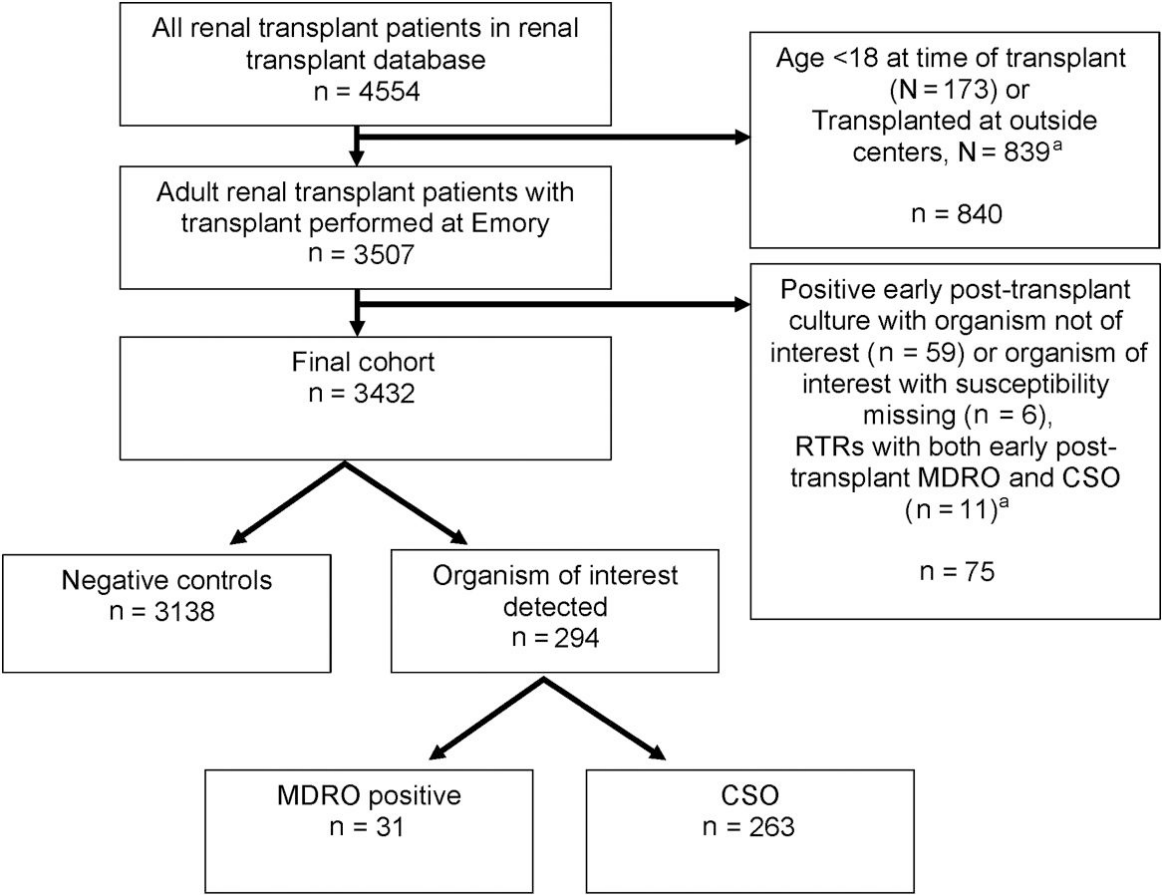
Study flow diagram. ^a^Groups not mutually exclusive. Abbreviations: CSO, comparative antibiotic-susceptible organism; MDRO, multidrug-resistant organism; RTR, renal transplant recipient.

Of the 4554 unique RTRs enrolled in the Emory Renal Transplant database between 2005 and 2021, 3507 met inclusion criteria (Figure 1). Among eligible RTRs (n = 3507), 328 (10.5%) had an EPT-positive culture with any organism. Eighty-three MDROs were detected among 44 unique RTRs, resulting in an EPT MDRO detection prevalence of 1.3% (95% CI, 0.91%–1.69%) (Supplementary Figure 3). ESCRE was the most common MDRO detected (52% [43/83]) followed by MRSA (25% [21/83]), VRE (14% [12/83]), CRPA (5% [4/83]), and CRAB (4% [3/82]). MDROs were most frequently detected in urinary tract samples (48% [40/83]), followed by blood (23% [19/83]), intra-abdominal specimens (18% [15/83]), respiratory tract samples (4% [3/83]), other invasive sites (4% [3/83]), and superficial wounds (2% [2/83]).

The incidence rate of EPT MDRO detection during the study period was 0.42 (95% CI, 0.31–0.57) per 1000 EPT-days at risk. The incidence during the pre-Enterobacterales cephalosporin and carbapenem breakpoint change period was 0.33 (95% CI, 0.14–0.71), which was similar to the incidence during the postbreakpoint change period (0.44; 95% CI, 0.31–0.61; *P* = .79). There was no association with time (year) in a negative binomial model adjusted for breakpoint change (*P* > .05) (Supplementary Table 2; Supplementary Figure 4).

### Impact of Early Post-transplant MDRO Detection on RTR Outcomes

Among eligible RTRs (n = 3507), 75 (2%, 75/3507) were excluded from survival analysis (Figure 1). Among RTRs who met survival analysis inclusion criteria (n = 3432), 91% (3138/3432) had no positive EPT cultures and were designated as negative controls, 8% (263/3432) had a CSO detected, and 1% (31/3432) had an MDRO detected in the EPT period (Figure 1, Table 1). Compared with negative controls, RTRs with positive EPT cultures were older (MDRO: median age [interquartile range {IQR}], 55 [43–64] years; CSO: median [IQR], 53 [43–64] years; negative controls: median [IQR], 50 [40–60] years), had a higher proportion of females (MDRO: 55% [17/31]; CSO: 62% [162/263]; negative controls: 41% [1274/3138]), had diabetes mellitus as the primary etiology of their ESRD (MDRO: 32% [10/31]; CSO: 38% [99/263]; negative controls: 30% [945/3138]), were dual organ transplant recipients (MDRO: 13% [4/31]; CSO: 9% [23/263]; negative controls: 7% [204/3138]), were deceased donor recipients (MDRO: 81% [25/31]; CSO: 76% [200/263]; negative controls: 67% [2107/3138]), had pretransplant MDRO detection (MDRO: 7% [2/31]; CSO: 3% [7/263]; negative controls: 2% [5/3138]), were on dialysis longer (MDRO: median [IQR], 7 [3.8–10.3] years; CSO: median [IQR], 4.7 [2.4–7.4] years; negative controls: median [IQR], 4.4 [2.2–7.3] years), and had a longer transplant admission length of stay (LOS; MDRO: median [IQR], 7 [4–15] days; CSO: median [IQR], 5 [4–8] days; negative controls: 4 [3–6] days). Remaining demographic and transplant-related characteristics are summarized in Table 1.

**Table 1.** Baseline Characteristics of Renal Transplant Recipients by Early Post-transplant Positive Culture Status, 2005–2021 (n = 3432)

Variables	Study Population, n = 3432	Negative Controls, n = 3138	Early Post-transplant Positive Culture, n = 294	
			CSO n = 263	MDRO, n = 31
Age, median [IQR], y	51 [40–60]	50 [40–60]	53 [43–63]	55 [46–63]
Sex, No. (%)				
Female	1453 (42)	1274 (41)	162 (62)	17 (55)
Race, No. (%)				
Black	1749 (51)	1607 (51)	128 (49)	14 (45)
White	1395 (41)	1271 (41)	109 (41)	15 (48)
Biracial	13 (0.4)	12 (0.4)	1 (0.4)	0 (0)
Other	252 (8)	226 (8)	4 (11)	22 (9)
Unknown	134 (4)	122 (4)	11 (4)	1 (3)
Primary ESRD etiology, No. (%)				
Diabetes mellitus	1054 (31)	945 (30)	99 (38)	10 (32)
Hypertension	858 (25)	796 (25)	49 (19)	13 (42)
Glomerular disease	665 (21)	611 (21)	4 (11)	50 (19)
Polycystic kidney disease	308 (9.0)	293 (9.3)	13 (5)	2 (7)
Other	494 (14)	443 (14)	48 (18)	3 (10)
Transplant type, No. (%)				
Kidney	3201 (93)	2934 (93)	240 (91)	27 (87)
Kidney-pancreas	229 (6.7)	202 (6)	23 (9)	4 (13)
Kidney-liver	2 (<0.01)	2 (<0.01)	0 (0)	0 (0)
Transplant year, No. (%)				
2005–2009	674 (20)	620 (20)	50 (19)	4 (13)
2010–2015	1188 (35)	1056 (34)	118 (45)	14 (45)
2016–2020	1570 (46)	1462 (47)	95 (36)	13 (42)
Donor type, No. (%)				
Deceased	2332 (68)	2107 (67)	200 (76)	25 (81)
Living	1100 (32)	1031 (33)	63 (24)	6 (19)
Years on dialysis, median [IQR]	4.4 [2.2–7.3]	4.4 [2.2–7.3]	4.7 [2.4–7.4]	7.0 [3.8–10.3]
Missing, No. (%)	1021 (29)	933 (30)	80 (30)	8 (26)
Immune induction protocol, No. (%)				
Basiliximab	2466 (72)	2256 (72)	188 (71)	22 (71)
Thymoglobulin	605 (18)	541 (17)	56 (21)	8 (26)
Other	361 (11)	341 (11)	19 (7)	1 (3)
CMV status, No. (%)				
D-/R-	402 (12)	376 (12)	260 (9)	3 (10)
D-/R+ or D+/R+	2454 (72)	2228 (72)	201 (77)	25 (83)
D+/R-	529 (16)	491 (16)	36 (14)	2 (7)
Missing	47 (1)	43 (1)	3 (1)	1 (3)
Rejection within first year post-transplant	441 (13)	399 (13)	38 (14)	4 (13)
MDRO detected pretransplant	67(2)	58(2)	7(3)	2(7)
Transplant admission LOS, median [IQR], d	4.0 [3.0–6.0]	4.0 [3.0–6.0]	5.0 [4.0–8.0]	7.0 [4.0–15.0]
Missing, No. (%)	1	1	0	0

Abbreviations: CSO, comparative antibiotic-susceptible organism; D, donor; ESRD, end-stage renal disease; IQR, interquartile range; LOS, length of stay; MDRO, multidrug-resistant organism; R, recipient.

Among RTRs with a positive EPT culture, the median (IQR) number of days between transplant and positive culture was 13 (7–20); this was similar across RTRs, with an MDRO and a CSO (median [IQR], 10 [18–18] days vs 13 [7–20] days; *P* = .40) detected. The most common organisms detected among RTRs were Enterobacterales (62% [183/294]), followed by *Enterococcus* spp. (24%, 72/294). The urinary tract (80%, 234/294) was the most common site of EPT-positive cultures, followed by blood (11%, 31/294). Trimethoprim-sulfamethoxazole resistance was common and similar among MDROs and CSOs in terms of which trimethoprim-sulfamethoxazole antimicrobial susceptibility testing is appropriate (59% [16/27] vs 66 [120/181]; *P* = .50) (Table 2).

**Table 2.** Microbiological Characteristics of Renal Transplant Recipients With Positive Early Post-transplant Cultures, 2005–2021 (n = 294)

Variables	Overall, n = 294	CSO, n = 263	MDRO, n = 31
Species, No. (%)^a^			
*Staphylococcus aureus*	12 (4)	6 (2)	6 (19)
*Enterococcus* spp.^b^	72 (24)	67 (25)	5 (16)
*Enterobacterales* ^c^	183 (62)	164 (62)	19 (61)
*Pseudomonas aeruginosa*	26 (9)	26 (10)	0 (0)
*Acinetobacter baumannii*	1 (0.3)	0 (0)	1 (3)
Trimethoprim-sulfamethoxazole resistance	136 (46)	120 (66)	16 (59)
Anatomic source, No. (%)^d^			
Urinary tract	234 (80)	214 (81)	20 (65)
Blood/endovascular	31 (11)	25 (10)	6 (19)
Respiratory	9 (3.1)	9 (3)	0 (0)
Superficial wound	3 (1.0)	3 (1)	0 (0)
Intra-abdominal	9 (3.1)	5 (2)	4 (13)
Superficial wound	3 (1)	3 (1)	0 (0)
Other invasive site	7 (2)	6 (2)	1 (3)
Stool	1 (0.3)	1 (0.4)	0 (0)
Time between transplant and positive culture, median [IQR], d	13 [7–20]	13 [7–20]	10 [8–18]

Abbreviations: CSO, comparative antibiotic-susceptible organism; IQR, interquartile range; MDRO, multidrug-resistant organism.

^a^If an RTR had multiple MDROs or susceptible organisms detected in the early post-transplant period, only the first organism was recorded.

^b^ *Enterococcus* spp. group includes *Enterococcus faecalis* (n = 65), *Enterococcus faecium* (n = 7).

^c^Enterobacterales group includes *Citrobacter freundii* complex (n = 8), *Enterobacter cloacae* complex (n = 7), *Enterobacter* spp. (n = 2)*, Escherichia coli* (n = 111), *Hafnia alvei* (n = 1), *Klebsiella aerogenes* (n = 3), *Klebsiella oxytoca* (n = 9), *Klebsiella pneumoniae (*n = 28), *Proteus mirabilis* (n = 8), *Providencia rettgeri* (n = 1), *Serratia marcescens* (n = 5). None were carbapenem resistant.

^d^Calculated for bacteria in which trimethoprim-sulfamethoxazole antimicrobial susceptibility testing is appropriate.

### Survival Analysis

**Figure 2.**
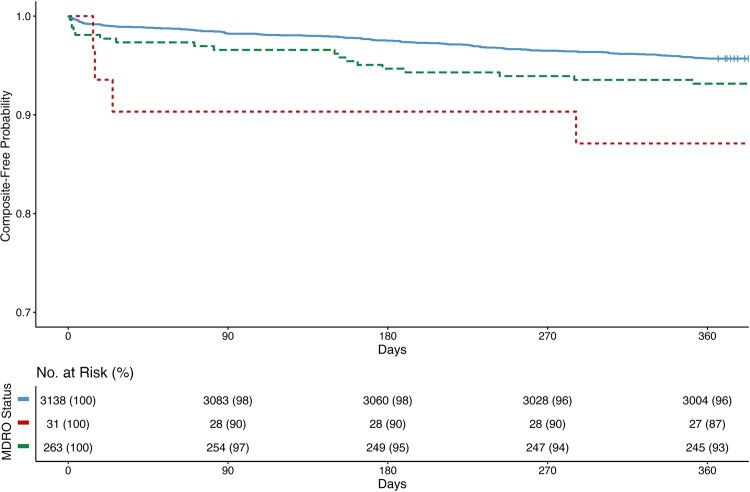
Kaplan-Meier analysis of composite outcome of renal transplant recipients by early post-transplant positive culture status, 2005–2021 (n = 3432). Kaplan-Meier analysis of composite outcome comparing renal transplant recipients with a multidrug-resistant organism detected on early post-transplant culture (red), a comparative antibiotic-susceptible organism detected on post-transplant culture (green), and negative controls (blue) (B). Time is measured from transplant until event. Log-rank *P* = .01. Abbreviation: MDRO, multidrug-resistant organism.

One hundred fifty-seven (5% [157/3432]) RTRs experienced the primary composite outcome. The median (IQR) number of days from transplant to primary composite outcome was 152 (24-235). There was a significant difference between time from transplantation to the composite outcome across the exposure categories (MDROs: median [IQR], 20 [15–90] days; CSO: median [IQR], 116 [8-174] days; negative controls: median [IQR], 155 [32–242] days; log-rank *P* = .01) (Figure 2). A higher proportion of RTRs in with MDRO EPT detection experienced the primary composite outcome compared with RTRs with CSO EPT detection and negative controls (MDRO: 13% [4/31]; CSO: 8% [18/263]; negative controls: 4% [135/3138]; *P* = .02). A higher proportion of RTRs with MDRO EPT detection had 1-year allograft failure compared with RTRs with CSO EPT detection and negative controls (10% [3/31] vs 3% [9/263] vs 2% [63/3138]; *P* = .01). One-year mortality was similar across the exposure categories (3% [1/31] vs 4% vs [11/263] vs 3% [86/3138]) (Supplementary Table 3).

The adjusted hazard ratio (aHR) of experiencing the primary composite outcome was higher among RTRs with EPT MDRO detection (aHR, 3.29; 95% CI, 1.21–9.92; *P* = .02) and EPT CSO detection (aHR, 1.54; 95% CI, 0.94–2.53; *P* = .09) compared with negative controls (Table 3). Similar trends were seen among RTRs with EPT MDRO detection in the postbreakpoint change subgroup (aHR, 3.46; 95% CI, 1.09–10.9; *P* = .04)) and kidney transplant–only subgroup (aHR, 2.70; 95% CI, 0.85–8.51; *P* = .09), but not among the urine-positive subgroup (aHR, 1.30; 95% CI, 0.18–9.39; *P* = .80). A similar trend was seen in the sensitivity analysis of RTRs with EPT MDRO detection in the MW cohort (aHR, 3.51; 95% CI, 1.26–9.78; *P* = .02) and IPTW cohort (aHR, 2.53; 95% CI, 0.94–6.83; *P* = .07) (Supplementary Table 4).

**Table 3.** Results of Cox Proportional Hazards Regression Model for Primary Outcome by Early Post-transplant Culture Positivity Status (n = 3432)

	Composite Outcome HR			
	HR (95% CI)	*P* Value	aHR (95% CI)^a^	*P* Value
Negative controls	Ref	-	Ref	-
CSO	1.62 (0.99–2.65)	.05	1.54 (0.94–2.53)	.09
MDRO	3.19 (1.18–8.63)	.02	3.29 (1.21–8.92)	.02

Abbreviations: aHR, adjusted hazard ratio; CSO, comparative antibiotic-susceptible organism; ESRD, end-stage renal disease; HR, hazard ratio; MDRO, multidrug-resistant organism.

^a^Adjusted for age, sex, year of transplant, 1-year post-transplant, deceased donor status, diabetes as the primary etiology of ESRD, and category of induction therapy.

### Competing Risk Analysis

**Figure 3.**
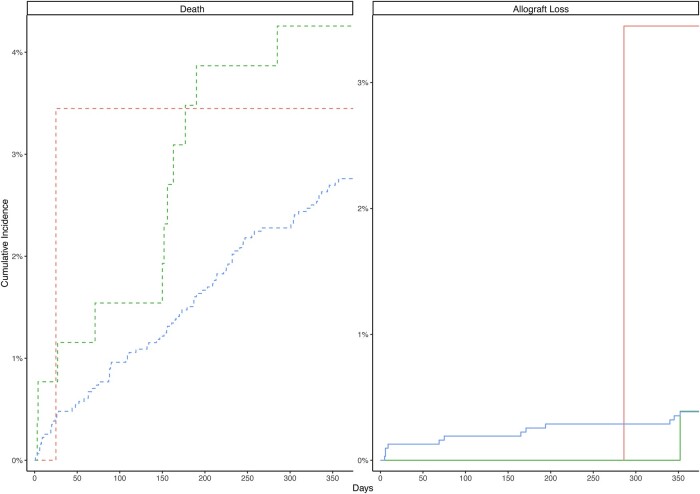
Cumulative incidence curves for 1-year mortality and 1-year allograft loss of renal transplant recipients by early post-transplant positive culture status, 2005–2021 (n = 3432). Cumulative incidence curves for 1-year mortality (left panel, dashed line) and 1-year allograft loss (right panel, solid line) comparing renal transplant recipients with a multidrug-resistant organism detected on early post-transplant culture (red), a comparative antibiotic-susceptible organism detected on post-transplant culture (green), and negative controls (blue). Time is measured from transplant until event.

Across the exposure categories, the cumulative incidence of 1-year allograft loss was significantly different across exposure groups (*P* = .04) (Figure 3; Supplementary Figure 5). When accounting for death as a competing risk, an increased adjusted cause-specific hazard (aHR, 7.15; 95% CI, 0.92–55.5; *P* = .06) and subdistribution hazard (aHR, 7.15; 95% CI, 0.95–53.7; *P* = .06) of 1-year allograft loss was observed among RTRs with EPT MDRO detection compared with negative control RTRs. This finding was not seen among RTRs with EPT CSO detection (cause-specific aHR, 0.84; 95% CI, 0.11–6.54; *P* = .9; subdistribution aHR, 0.84; 95% CI, 0.12–6.03; *P* = .9) (Table 4).

**Table 4.** Competing Risk Analysis Results Stratified by Early Post-transplant Positive Culture Status (n = 3432)

	Cause-Specific			Subdistribution		
	One-Year Mortality			One-Year Mortality		
	HR (95% CI)	aHR^a^ (95% CI)	*P* Value*	HR (95% CI)	aHR^a^ (95% CI)	*P* Value*
Negative controls	Ref	Ref	-	Ref	Ref	-
CSO	1.56 (0.83–2.92)	1.38 (0.73–2.59)	.3	1.56 (0.83–2.93)	1.38 (0.72–2.64)	.30
MDRO	1.26 (0.18–9.08)	1.34 (0.19–9.66)	.8	1.26 (0.17–9.26)	1.34 (0.18–10.1)	.80

…	Cause-Specific			Subdistribution		
…	One-Year Allograft Loss			One-Year Allograft Loss		
…	HR (95% CI)	aHR^a^ (95% CI)	*P* Value	HR (95% CI)	aHR^a^ (95% CI)	*P* Value
Negative controls	Ref	Ref	-	Ref	Ref	…
CSO	1.02 (0.13–7.82)	0.84 (0.11–6.54)	.9	1.02 (0.13–7.79)	0.84 (0.12–6.03)	.9
MDRO	9.41 (1.16 68.8)	7.15 (0.92–55.5)	.06	8.95 (1.18–67.9)	7.15 (0.95–53.7)	.06

Abbreviations: aHR, adjusted hazard ratio; CSO, comparative antibiotic-susceptible organism; ESRD, end-stage renal disease; HR, hazard ratio; MDRO, multidrug-resistant organism.

^a^Adjusted for age, sex, year of transplant, deceased donor status, diabetes as the primary etiology of ESRD, and category of induction therapy.

^*^ *P* value of adjusted model.

Similar results were seen among RTRs with EPT MDRO detection in the postbreakpoint change subgroup (cause-specific aHR, 9.98; 95% CI, 1.26–79.2; *P* = .03), the urine-positive subgroup (cause-specific aHR, 10.1; 95% CI, 1.28–79.9; *P* = .03), the kidney transplant–only subgroup (aHR, 7.44; 95% CI, 0.95–58.3; *P* = .06), RTRs in the MW cohort (cause-specific aHR, 15.1; 95% CI, 1.61–142.00; *P* = .02), and RTRs in the IPTW cohort (cause-specific aHR, 7.18; 95% CI, 0.97–53.3; *P* = .05) (Supplementary Table 5).

## DISCUSSION

Our study of >3500 RTRs over a 16-year time period represents one of the largest studies to estimate the burden of MDRO detection during this critical EPT period and to examine MDRO detection the association of MDRO EPT detection with key outcomes. We found the incidence and prevalence of EPT MDRO detection to be low. However, MDRO detection during the EPT period was significantly associated with an increased hazard of 1-year mortality or allograft loss (the composite primary outcome) and of death-censored allograft loss, adjusting for important variables when compared with negative control RTRs. These findings were robust across the various subgroup and PS-weighted sensitivity analyses.

The first 30 days in the post-transplant period represent a time when RTRs are most vulnerable to nosocomial MDRO infection [26–28]. We found MDRO culture positivity during this period predominated by uropathogenic bacteria. Our findings are in concordance with the epidemiology described in other large cohort studies [27, 29] and reflects the practice of frequent urine sampling [30]. Consistent with prior literature, RTRs with EPT MDROs were more frequently of older age, female, diabetic, recipients of a kidney from a deceased donor, and had a longer transplant admission length of stay [31, 32]. While our prevalence and incidence of MDRO detection were low, these findings likely represent the tip of the iceberg, as clinical culture positivity underestimates rates of MDRO colonization compared with active surveillance [33]. Hence, further prospective active surveillance studies to determine the rates of MDRO colonization among RTRs are needed [9].

We found that EPT MDRO detection resulted in an increased hazard of the composite outcome of 1-year death or allograft loss, which was driven by an increase in death-censored allograft loss. A similar signal was seen among RTRs with a CSO detected. Prior studies have previously demonstrated poor outcomes in RTRs with MDRO infections [4]. Our study builds on previous findings by examining the specific impact of MDRO detection, regardless of symptoms (ie, inclusive of those with MDRO colonization), among a large cohort during the critical EPT period [27]. Our cohort was primarily comprised of RTRs with positive urinary tract cultures. Among this subgroup, we found an increased hazard of death-censored allograft loss but not death, likely due to the removal of those with bloodstream infections. Bacterial invasion of the urinary tract in RTRs is hypothesized to lead to an inflammatory response and cytokine activation, contributing to allograft dysfunction [34]. The association of allograft loss and urinary tract infections (UTIs) and/or asymptomatic bacteriuria (ASB) in the EPT remains undetermined [14]. Most prior studies included patients with UTI/ASB in the post–early transplant (after 30 days) period or had a wide inclusion period [30, 35]. The lack of association in the CSO group suggests the additive impact of antimicrobial resistance on RTR outcomes [4, 5]. This may be due to different treatment regimens of RTRs with a MDRO vs CSO detected. Current AST recommendations do not endorse the routine treatment of ASB [30]. Multicenter studies using standardized definitions to parse the impact of ASB and UTI on graft function and to guide management are required. In such studies, stratification based on susceptibility profile or adjusting for inappropriate empiric therapy will be helpful to assess the impact of resistance on outcomes [36].

Increased stewardship and infection prevention–based interventions to decrease MDRO acquisition in ESRD and RTRs are of importance. These include both system- and patient-level interventions such as limiting the duration of urinary catheters and stents, avoiding broad-spectrum nephrotoxic agents when possible, and vigilance in standard infection prevention practices [37–39]. Pretransplant MDRO infection/colonization has been associated with high rates of EPT MDRO invasive infection [3, 40, 41]. If our findings are replicated in prospective studies, active surveillance of RTRs for MDROs before transplantation and decolonization using novel techniques such as microbiome therapeutics may be considered in the future [42, 43]. We recently demonstrated the safety and efficacy of fecal microbiota transplantation (FMT) in a phase 1 randomized controlled trial of FMT administered for MDRO decolonization among RTRs [44]. Moreover, in a post hoc analysis, the FMT-treated participants in that trial had a longer time to recurrent MDRO infection compared with matched RTRs who met FMT eligibility criteria but were not treated with FMT.

While our study builds on the limitations of prior studies in terms of sample size, the expanded inclusion of multiple MDROs, and time periods analyzed, some key limitations exist. First, our study is an observational study with relatively infrequent MDRO positivity, and thus subject to residual confounding. While we attempted to account for confounding by multivariable adjustment and PS-weighted sensitivity analysis, important potential confounders were not available in the data set. Of greatest importance is the lack of antibiotic exposure data in the EPT period as these may have driven the difference in outcomes seen among the exposure groups through inappropriate empiric therapy or increased exposure to nephrotoxic agents [36]. Other important variables that were unavailable include severity of illness, intensive care unit admission, timing of ureteral stent removal, postoperative complications, and intraoperative variables such as cold ischemic time and need for surgical re-exploration. Second, we were unable to use raw minimal inhibitory concentration data for our classification of MDROs, which may lead to misclassification bias due to changes in MDRO definitions over time. To account for this and for clinical practice changes over time, we included transplantation year as an adjustment variable in the model and in the PS creation. Moreover, the results of the subgroup analysis of the postbreakpoint change cohort were similar to our primary findings. Third, like prior studies [39, 45], our case definition was based on culture positivity in the absence of symptoms or clinical indicators of infection. While this may be a reflection of severity and/or chronicity of underlying illness and could be considered a surrogate for health care exposure, we believe that culture positivity (colonization) represents an important dysbiotic phenotype associated with poor outcomes and subsequent invasive infections [40–42, 46]. Fourth, our MDRO definitions are not inclusive of all clinically relevant MDRO phenotypes such as carbapenem-susceptible multidrug-resistant *Pseudomonas* and other difficult-to-treat resistant phenotypes [47, 48]. Finally, as center-level characteristics are associated with RTR outcomes, our findings in a single center may not be generalizable to other centers and require further validation in multicenter studies [49].

Overall, our findings reveal that the acquisition or presence of MDROs in this early post-transplant period, while infrequent, was associated with the study primary composite outcome. These results underline the need for continued studies to elucidate causal pathways and confirm our findings. If replicated, these findings call for increased stewardship and infection prevention efforts in this vulnerable population.

## Supplementary Material

ofae060_Supplementary_Data

## Supplementary Data

Supplementary materials are available at *Open Forum Infectious Diseases* online. Consisting of data provided by the authors to benefit the reader, the posted materials are not copyedited and are the sole responsibility of the authors, so questions or comments should be addressed to the corresponding author.
