# Correction to: The Burden and Impact of Early Post-transplant Multidrug-Resistant Organism Detection Among Renal Transplant Recipients, 2005–2021

**DOI:** 10.1093/ofid/ofae541

**Published:** 2024-09-20

This is a correction to: Ahmed Babiker, Geeta Karadkhele, Andrei Bombin, Rockford Watkins, Chad Robichaux, Gillian Smith, Vivek B Beechar, Danielle B Steed, Jesse T Jacob, Timothy D Read, Sarah Satola, Christian P Larsen, Colleen S Kraft, Stephanie M Pouch, Michael H Woodworth, The Burden and Impact of Early Post-transplant Multidrug-Resistant Organism Detection Among Renal Transplant Recipients, 2005–2021, *Open Forum Infectious Diseases*, Volume 11, Issue 3, March 2024, ofae060, https://doi.org/10.1093/ofid/ofae060

In the originally published version of this manuscript, there was a typographical error in the name of the ninth-listed author, Jesse T. Jacob.

This has been corrected.

