# Supplementary material for: The Burden and Impact of Early Post-transplant Multidrug-Resistant Organism Detection Among Renal Transplant Recipients, 2005–2021
Source: Open Forum Infect Dis. 2024 Feb 13;11(3):ofae060. doi: 10.1093/ofid/ofae060 (PMC10924447; doi:10.1093/ofid/ofae060)
Supplement: ofae060_Supplementary_Data [file ofae060_supplementary_data.docx]

**The Burden and Impact of Early Post-Transplant Multidrug-resistant Organism Detection Among Renal Transplant Recipients, 2005-2021**

**Supplementary Material**

*Propensity Score Weighted Cohorts Generation*

The Propensity Score (PS) was created by performing a multinomial logistic regression model which modeled the exposure category (negative controls renal transplant recipients [RTRs], RTRS with an early post-transplant [EPT] comparative antibiotic susceptible organism [CSO] detected and RTRs with an EPT multi-drug resistant organism [MDRO] detected) as the outcome variable with backward variable selection for the final model selection. Age at transplant, sex, category of induction regimen, type of donor, and transplant year were all associated with the MDRO or CSO detection in the EPT period and were included in the PS generation, After generation of the PS, two methods of weighting, inverse probability of treatment weighting (IPTW) and matching weighting (MW), which incorporated the PS were applied to create weighted cohorts ^1^. PS covariate balance before and after generation of the weighted cohorts were assessed by examining the standard mean difference for variables across the exposure category (**Supplementary Material,** **Supplementary Figure 2**). PS weighting resulted in better covariate balance among the MW cohort compared to IPTW cohort (**Supplementary Figure 2**).

*Statistical Analysis*

The prevalence of EPT MDRO detection was estimated using all eligible RTRs as the denominator. The incidence rate of EPT MDRO detection was calculated per 1,000 EPT-days at risk . At-risk time was calculated for each RTR as time between transplantation and positive early MDRO culture (event), allograft loss or death or 30 days (whichever came first). Differences in pre- and post-breakpoint change incidence rates were assessed using Wilcoxon signed-rank test. Overall change in incidence over time was assessed using a negative binomial model with adjustment for pre- and post- breakpoint change period and model fit was assessed using log likelihood test (compared to model without breakpoint change).

**Supplementary Figures**

**Supplementary Figure 1**. Study Directed Acyclic Graph

Abbreviations: CMV: cytomegalovirus, ICU: Intensive care unit, MDRO: multidrug resistant organism

# Supplementary Figure 2. Standardized mean differences for key covariates across weighting methods.

**Supplementary Figure 2.** Standardized mean differences for key covariates which included the propensity score generation averaged across three exposure categories in the unmatched (red, solid line), IPTW (green, dotted line) and matching weights (blue, dashed line), cohorts. Matching weights achieved better covariate balance than IPTW.

#

**Supplementary Figure 3**. Waffle plot of all RTRS (N=3,507) stratified by early post-transplant culture status, 2005-2021


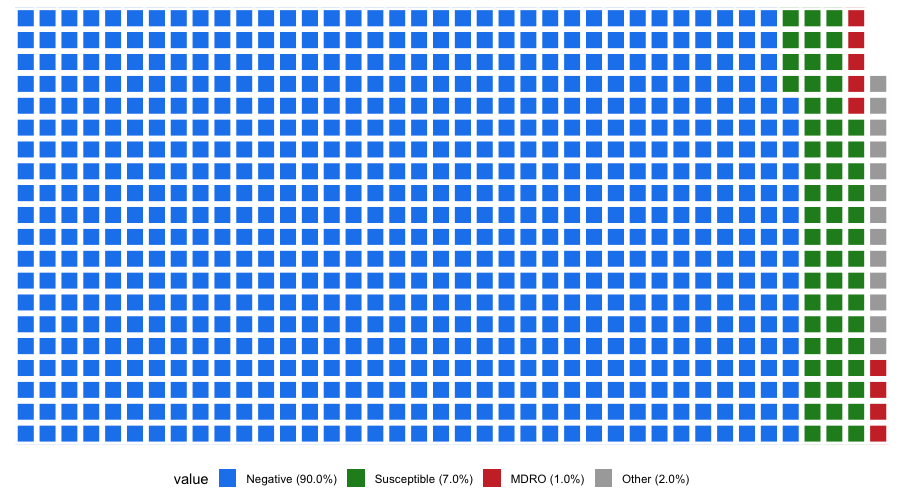


**Supplementary Figure 3 legend**. Waffle plot of all RTRS (N=3,507) stratified by early post-transplant culture status into those with no early-post transplant culture positivity (blue), those with early-post transplant culture positive with a target MDRO (red), a comparative antibiotic susceptible organism (blue) or a non-target organism (grey).

**
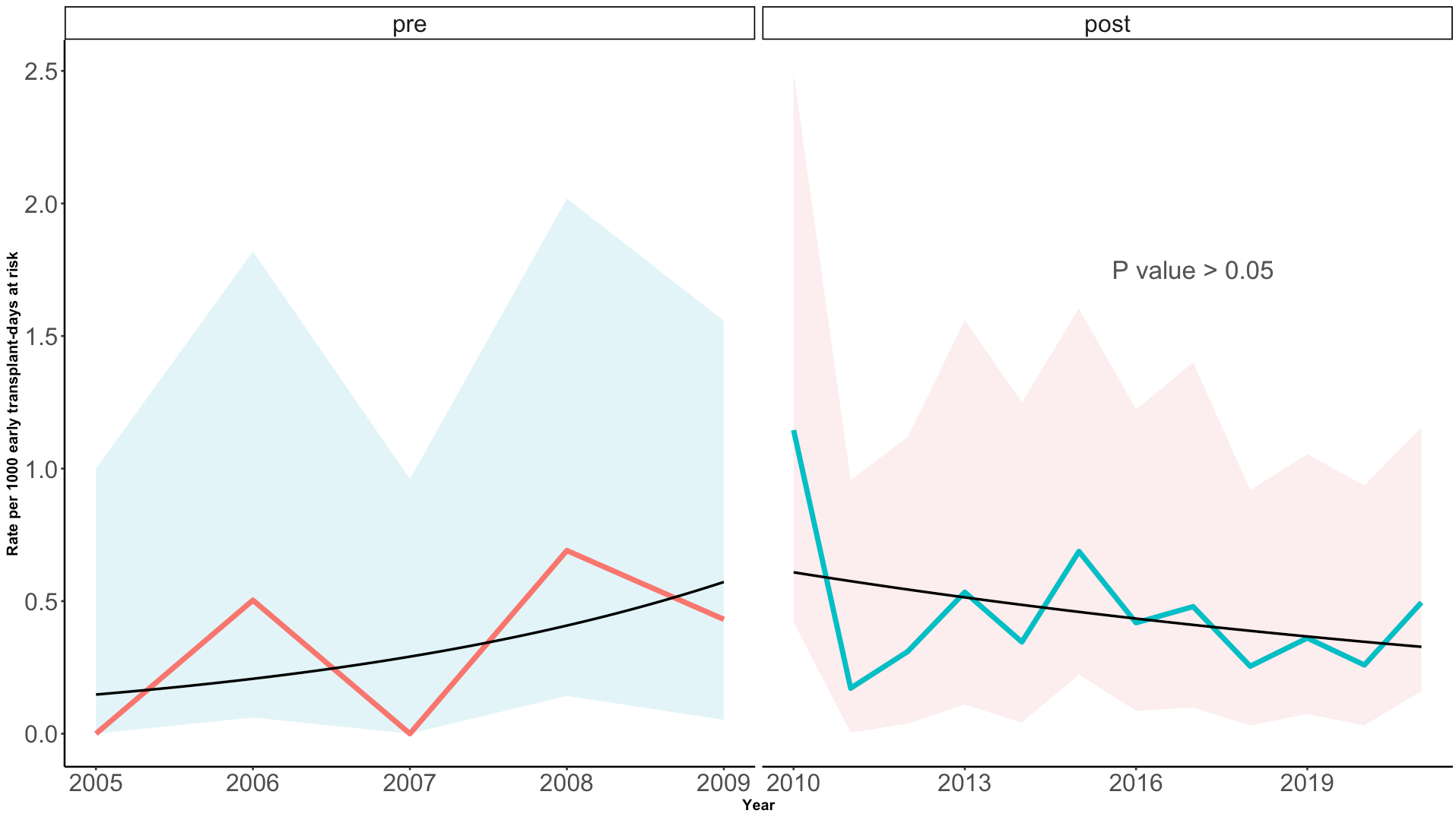
Supplementary Figure 4.** Incidence of Multi-drug resistant organism detection per 1000 early post-transplant days, 2005-2021

**Supplementary Figure 4**. Incidence of MDRO detection per 1000 early post-transplant days at risk in the pre- (left panel) and post- (right panel) Enterobacterales breakpoint change period. 95% confidence interval represented by shaded areas and line of best fit (black) added using binomial regression. *P-value* for binomial regression model examining association of incidence with time (year) adjusting for breakpoint change period.

**Supplementary Figure 5.** Cumulative incidence curves for one-year mortality and one-year allograft loss of Renal Transplant Recipients by Early Post Transplant Positive Culture status, 2005-2021 (N= 3,432)

**Supplementary Figure 5 Legend**. Cumulative incidence curves for one-year mortality (dashed line) and one-year allograft loss (solid line) comparing renal transplant recipients with a multi-drug resistant organism detected on early post-transplant culture (red), a comparative antibiotic susceptible organism detected on post-transplant culture (green) and negative controls (blue). Time is measured from transplant until event.

Abbreviations: CSO: comparable susceptible organism, MDRO: multidrug-resistant organism

**Supplementary Tables**

**Supplementary Table 1:** Target Study Multidrug-resistant organisms and Comparative antibiotic susceptible organisms

| **Multidrug-resistant organisms** | **Comparative antibiotic susceptible organisms** |
| --- | --- |
| Methicillin resistant *Staphylococcus aureus* | Methicillin resistant *Staphylococcus aureus* |
| Vancomycin resistant *Enterococcus* | Vancomycin susceptible *Enterococcus* |
| Extended-spectrum cephalosporin resistant Enterobacterales | Extended cephalosporin susceptible Enterobacterales |
| Carbapenem-resistant Enterobacterales | n/a |
| Carbapenem-resistant *Acinetobacter baumanii* complex | Carbapenem susceptible *Acinetobacter baumanii* complex |
| Carbapenem-resistant *Pseudomonas aeruginosa* | Carbapenem-susceptible *P. aeruginosa* |

Antimicrobial susceptibility testing was performed at the Emory University Hospital microbiology laboratory as part of routine clinical care on the Vitek 2 (bioMérieux, Durham, NC, USA) and MicroScan (Beckman Coulter, Brea, CA) platforms. Multidrug resistant organisms and comparative antibiotic susceptible organisms were defined as per Clinical and Laboratory Standards Institute breakpoints each year ^2^.

**Supplementary Table 2:** Early Post-Transplant Multidrug-resistant Organism Prevalence and Incidence per Year, 2005-2021

| **Year** | **Number of renal transplants performed** | **EPT MDRO positive cultures** | **Incidence per 1000 EPT days at risk [95 CI]** |
| --- | --- | --- | --- |
| 2005 | 120 | 0 | 0.00 [0.00, 1.00] |
| 2006 | 136 | 2 | 0.50 [0.06, 1.81] |
| 2007 | 128 | 0 | 0.00 [0.00, 0.96] |
| 2008 | 147 | 3 | 0.69 [0.14, 2.02] |
| 2009 | 157 | 2 | 0.43 [0.05, 1.56] |
| 2010 | 178 | 6 | 1.15 [0.42, 2.49] |
| 2011 | 195 | 1 | 0.17 [0.00, 0.96] |
| 2012 | 218 | 2 | 0.31 [0.04, 1.12] |
| 2013 | 193 | 3 | 0.53 [0.11, 1.56] |
| 2014 | 194 | 2 | 0.35 [0.04, 1.25] |
| 2015 | 246 | 5 | 0.69 [0.22, 1.60] |
| 2016 | 242 | 3 | 0.42 [0.09, 1.22] |
| 2017 | 210 | 3 | 0.48 [0.10, 1.40] |
| 2018 | 264 | 2 | 0.25 [0.03, 0.92] |
| 2019 | 280 | 3 | 0.36 [0.07, 1.05] |
| 2020 | 259 | 2 | 0.26 [0.03, 0.94] |
| 2021 | 340 | 5 | 0.50 [0.16, 1.16] |

Abbreviations: CI: confidence interval, EPT: early post-transplant, MDRO: multidrug-resistant organism

# Supplementary Table 3: Outcomes of Renal Transplant Recipients Stratified by Early Post Transplant Positive Culture Status, 2005-2021 (N= 3,432)

| **Variables** | **Study Population,**  **N= 3,432** | **Negative Controls,**  **N= 3,138** | **Early Post Transplant Positive Culture,**  **N=294** | |
| --- | --- | --- | --- | --- |
|  |  |  | **CSO**  **N= 263** | **MDRO,**  **N = 31** |
| One-year post-transplant mortality, N (%) | 98 (2.9) | 86 (2.7) | 11 (4.2) | 1 (3.2) |
| One-year post-transplant graft failure, N (%) | 75 (2.2) | 63 (2.0) | 9 (3.4) | 3 (9.7) |
| Composite Outcome, N (%) | 157 (4.6) | 135 (4.3) | 18 (7.6) | 4 (13) |

Abbreviations: CSO: comparative antibiotic susceptible organism, MDRO: multidrug-resistant organism

# Supplementary Table 4. Results of Sensitivity and Subgroup Analysis of Cox Proportional Hazards of Composite Outcome among RTRs

| Cohort |  |  | **Composite outcome HR** | |  |
| --- | --- | --- | --- | --- | --- |
|  |  | HR (95%CI) | *P-*value | aHR (95%CI)* | *P-*value |
| MW cohort  (N=3,432) | Negative controls (N=3,138) | Ref | - | Ref | - |
|  | CSO (N=263) | 1.86 (1.11, 3.12) | 0.02 | 1.89 (1.11, 3.20) | 0.02 |
|  | MDRO (N=31) | 3.27 (1.21, 8.82) | 0.01 | 3.51 (1.26, 9.78) | 0.02 |
| IPTW cohort (N=3,432) | Negative controls (N=3,138) | Ref | - | Ref | - |
|  | CSO (N=263) | 1.61 (0.93, 2.78) | 0.09 | 1.53 (0.86, 2.70) | 0.20 |
|  | MDRO (N=31) | 2.74 (0.97, 7.79) | 0.06 | 2.53 (0.94, 6.83) | 0.07 |
| Urine subgroup  (N= 3,372) | Negative controls (N=3,138) | Ref | - |  | - |
|  | CSO (N=214) | 0.76 (0.35, 1.61) | 0.5 | 0.73 (0.34, 1.58) | 0.40 |
|  | MDRO (N=20) | 1.15 (0.16, 8.20) | 0.9 | 1.30 (0.18, 9.39) | 0.80 |
| Post Breakpoint subgroup  (N=2,585) | Negative controls (N=2,364) | Ref | - |  |  |
|  | Susceptible organism (n=199) | 1.79 (1.02, 3.14) | 0.04 | 1.74 (0.99, 3.07) | 0.06 |
|  | MDRO (n=22) | 3.60 (1.14, 11.4) | 0.03 | 3.46 (1.09, 10.9) | 0.04 |
| Renal Transplant Only subgroup  (N= 3,201) | Negative controls (N=2,934) | Ref | - |  |  |
|  | Susceptible organism (n=240) | 1.74 (1.04, 2.87) | 0.04 | 1.58 (0.95, 2.64) | 0.08 |
|  | MDRO (n=27) | 2.77 (0.88, 8.72) | 0.08 | 2.70 (0.85, 8.51) | 0.09 |

Abbreviations: aHR: adjusted hazards ratio, CSO: comparative antibiotic susceptible organism, HR: hazards ratio, MDRO: multidrug-resistant organism

*Adjusted for age, sex, year of transplant, deceased donor status, diabetes as the primary etiology of ESRD and category of induction therapy

# Supplementary Table 5. Cause specific Hazards of Allograft loss among subgroup and sensitivity analysis cohorts.

|  |  | **Cause Specific**  **One year Allograft Loss** | | |
| --- | --- | --- | --- | --- |
| Cohort |  |  |  |  |
|  |  | *HR (95%CI)* | *aHR*(95%CI)* | *P-*value ** |
| MW cohort  (N=3,432) | Negative controls (N=3,138) | Ref | Ref | - |
|  | CSO (N=263) | 1.10 (0.14, 8.61) | 1.17 (0.14, 9.63) | 0.9 |
|  | MDRO (N=31) | 7.67 (1.01, 58.5) | 15.1 (1.61, 142.0) | 0.02 |
| IPTW cohort (N=3,432) | Negative controls (N=3,138) | Ref | Ref | - |
|  | CSO (N=263) | 0.43 (0.06, 3.28) | 0.56 (0.06, 5.05) | 0.6 |
|  | MDRO (N=31) | 6.23 (0.81, 47.8) | 7.18 (0.97, 53.3) | 0.05 |
| Urine subgroup (N= 3,372) | Negative controls (N=3,138) | Ref | Ref | - |
|  | CSO (N=214) | 1.21 (0.16, 9.34) | 1.01 (0.13, 7.86) | >0.9 |
|  | MDRO detected (N=20) | 13.0 (1.69, 99.7) | 10.1 (1.28, 79.9) | 0.03 |
| Post Breakpoint subgroup  (N=2,585) | Negative controls (N=2,364) | Ref | Ref | - |
|  | CSO (n=199) | 0.00 (0.00, ∞) | 0.00 (0.00, ∞) | >0.9 |
|  | MDRO detected (n=22) | 11.3 (1.45, 88.3) | 9.98 (1.26, 79.2) | 0.03 |
| Renal Transplant Only subgroup  (N= 3,201) | Negative controls (N=2,934) | Ref | Ref | - |
|  | Susceptible organism (n=240) | 1.14 (0.15, 8.81) | 0.89 (0.11, 6.95) | >0.9 |
|  | MDRO (n=27) | 10.3 (1.33, 79.9) | 7.44 (0.95, 58.3) | 0.06 |

Abbreviations: aHR: adjusted hazards ratio, CSO: comparative antibiotic susceptible organism, HR: hazards ratio, MDRO: multidrug-resistant organism

*Adjusted for age, sex, year of transplant, deceased donor status, diabetes as the primary etiology of ESRD and category of induction therapy

** *p-*value of adjusted model

**References**

1. Li L, Greene T. A weighting analogue to pair matching in propensity score analysis. *Int J Biostat.* Jul 31 2013;9(2):215-234.

2. Clinical and Laboratory Standards Institute. M100-S27 Psfast, 27th informational supplement. Wayne, PA: Clinical and Laboratory Standards Institute; 2017. *Clinical and Laboratory Standards Institute. M100-S27, Performance standards for antimicrobial susceptibility testing, 27th informational supplement. Wayne, PA: Clinical and Laboratory Standards Institute; 2017.* 2017.
